# Validation of a Natural Language Processing Algorithm for the Extraction of the Sleep Parameters from the Polysomnography Reports

**DOI:** 10.3390/healthcare10101837

**Published:** 2022-09-22

**Authors:** Mahbubur Rahman, Sara Nowakowski, Ritwick Agrawal, Aanand Naik, Amir Sharafkhaneh, Javad Razjouyan

**Affiliations:** 1 Houston Veterans Affairs Health Services Research and Development Service, Center for Innovations in Quality, Effectiveness and Safety, Michael E. DeBakey Veteran Affairs Medical Center, Houston, TX 77030, USA; 2 Department of Medicine, Baylor College of Medicine, Houston, TX 77030, USA; 3 Medical Care Line, Michael E. DeBakey Veteran Affairs Medical Center, Houston, TX 77030, USA; 4 Veterans Affairs South Central Mental Illness Research, Education and Clinical Center, Houston, TX 77030, USA; 5 University of Texas School of Public Health, 1200 Pressler Str., Houston, TX 77030, USA

**Keywords:** polysomnography, natural language processing, sleep parameters

## Abstract

Background: There is a need to better understand the association between sleep and chronic diseases. In this study we developed a natural language processing (NLP) algorithm to mine polysomnography (PSG) free-text notes from electronic medical records (EMR) and evaluated the performance. Methods: Using the Veterans Health Administration EMR, we identified 46,093 PSG studies using CPT code 95,810 from 1 October 2000–30 September 2019. We randomly selected 200 notes to compare the accuracy of the NLP algorithm in mining sleep parameters including total sleep time (TST), sleep efficiency (SE) and sleep onset latency (SOL), wake after sleep onset (WASO), and apnea-hypopnea index (AHI) compared to visual inspection by raters masked to the NLP output. Results: The NLP performance on the training phase was >0.90 for precision, recall, and F-1 score for TST, SOL, SE, WASO, and AHI. The NLP performance on the test phase was >0.90 for precision, recall, and F-1 score for TST, SOL, SE, WASO, and AHI. Conclusions: This study showed that NLP is an accurate technique to extract sleep parameters from PSG reports in the EMR. Thus, NLP can serve as an effective tool in large health care systems to evaluate and improve patient care.

## 1. Introduction

Polysomnography (PSG) uses multiple parameters to evaluate and diagnose sleep disorders [1]. PSG is considered the gold standard for diagnosing sleep disorders and includes the measurement of several physiological activities, such as brain waves via electroencephalography (EEG), heart rate via electrocardiogram (EKG), eye movements via electrooculogram (EOG), muscle movements via electromyography (EMG), nasal and oral airflow via thermistor/thermocouple and through nasal pressure changes, respiratory effort via abdominal and thoracic bands, and blood oxygen saturation via pulse oximetry [2]. The PSG reports document several components such as patient demographic information, technical detail regarding the physiological records (e.g., number of electrodes), sleep continuity (e.g., total sleep time), sleep stages architecture (e.g., NREM Stage 1), respiratory indices (e.g., apnea hypopnea index [AHI]), and periodic limb movements (e.g., PLMI) [3]. The PSG report’s components are typically stored as a free-text note in the electronic medical record (EMR), which is not readable, processable, or computable and is considered unstructured data [4]. 

To convert unstructured data into structured data that is readable, processable, and computable data, researchers use various methods [5,6,7]. These methods include a traditional approach via manual reviewing of notes and converting them into the structured data. The manual method is time-consuming, not feasible for a large quantity of notes, and prone to human error [8]. More recently, the use of novel machine learning techniques, such as natural language processing (e.g., NLP) algorithms, has been proposed to solve the shortcomings of manual data extractions. NLP has been successfully implemented within the Veterans Health Administration (VHA) EMR to extract data from clinical notes for ejection fraction and heart failure [9,10,11]. There are several other clinical studies using NLP algorithm on the VHA EMR database [12,13,14,15]. Few studies have reported the use of NLP to convert unstructured data from PSG reports into structured data [16,17]. Investigators used regular expression matching techniques to extract total sleep time (TST) with an accuracy of 80%; however, they did not provide detail on validation information in the published study. In addition, they did not extract the part of text to analysis the data and it limited the performance of their algorithm. 

The aim of the present study was to develop an NLP algorithm based on prior work [18] to extract sleep continuity parameters including total sleep time (TST), sleep efficiency (SE), sleep onset latency (SOL), wake after sleep onset (WASO), and respiratory index (AHI) from PSG reports in the VHA EMR and to test the accuracy of the NLP algorithm compared to annotators/raters masked to NLP output.

## 2. Methods

In this study, we used the Corporate Data Warehouse [19]. The study protocol was approved by the Research & Development Committee of the Michael E. DeBakey VA Medical Center and Baylor College of Medicine Institutional Review Board (IRB# H-35366).

### 2.1. Cohort

This is a retrospective study utilizing the VHA EMR from 1 October 1999, through 30 September 2020. We included patients who had any International Classification of Disease, 9th edition (ICD-9) or 10th edition (ICD-10) sleep disease, Supplementary Table S1. The VA EMR is also known as the VHA’s Corporate Data Warehouse (CDW). It is a relational database that collects veterans data from all VHA facilities from October 1999 to the present [20].

### 2.2. Database

The cohort was selected from 4,237,444 patients. We limited the results to the patients who had the following Current Procedural Terminology (CPT) procedure codes: 95810 and 95811. We included patients’ notes that were associated with visits with the CPT codes had “%poly%” OR “%PSG%” in the title of notes; had “%PSG%” OR “%polysomno%” in the body of notes; and did not have “%titrat%” OR “%split%” in the body of text, which represented split-night studies.

For the remaining notes, we developed a document quality score, which we refer to as a document quality index (DQI). The DQI is a score ranging from zero to seven. Zero referred to no sleep parameter phrases used in the note, and seven referred to full documentation of the sleep parameters. The DQI was calculated by summing seven components, and each component related to a sleep parameter. We assigned zero to each component if the sleep parameter of interest was not documented. The components were as follows: (1) TST, “%TST%” or “%total sleep time%”; (2) SoL, “%SoL%” or “%onset latency%”; (3) SE, “%SE%” or “%sleep efficiency%”; (4) WASO, “%WASO%” or “%wake after sleep onset%”; (5) rapid eye movement (REM), “%REM%”; (6) sleep stage 1 (N1), “%N1%”; (7) AHI, “%AHI%” or “%apnea%index%”. After consulting with the team of sleep board-certified physicians (AS and RA) providing them with samples of notes with the scores greater than 3, the medical team recommended using a cut of 4 or greater. We had 46,093 notes from 42,991 patients.

### 2.3. Sampling Strategy for the NLP Development and Reference Standard

We applied the exact power calculation method to determine the minimum required sample size for the validation of the NLP algorithm using the recommend sampling strategy [21]. With assumption of effect size of 0.3, alpha of 0.05, power of 80, and degree of freedom of 5, the minimum number of notes for each variable was 143. However, the annotators reviewed 200 randomly selected notes, with 160 notes for the training performance and 40 notes for the validation performance of the NLP algorithm. Two board-certified sleep medicine specialists reviewed and labeled the sampled generated data for validation of the NLP algorithm (AS and RA). In case of discrepancy, the final decision was judicated liberally by adding another SN as final voter. The two raters were blinded from each other. During the judication process of discrepancy, the raters informed about their labels. Additionally, we used the Cohen’s κ, Intraclass correlation coefficient (ICC) [22,23] to understand the level of reviewers’ agreement. The ICC ranges from zero (no agreement) to one (perfect agreement). The ICC was interpreted as: poor (<0.5), moderate (0.5–0.7), good (0.75–0.9), and excellent (≥0.9).

### 2.4. NLP Algorithm Development for the Extraction of the Sleep Parameters

**Figure 1:**
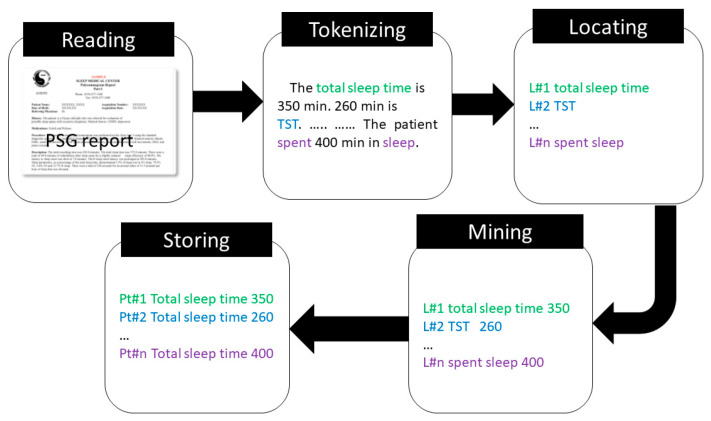
An NLP flow diagram to extract sleep parameters from the PSG reports.

We developed an NLP algorithm for each sleep parameter of interest. Therefore, the following NLP algorithm for extracting the sleep continuity parameters was repeated for each sleep parameter, Figure 1. The NLP algorithm receives the PSG reports as input texts and generates the sleep parameter associated with the respected quantity along with the patient’s identification and visit date to a structured output format. We divided the NLP algorithm into five steps (Figure 1). The algorithm was developed with the Python programming language [24]. We used the Natural Language Toolkit (NLTK), which is a suite of libraries and program to process human language data [25].

1. Reading: Read the PSG reports and store in the memory.
2. Tokenizing: Tokenize the PSG reports.
3. Locating: Identify the location of the sleep parameter of interest.
4. Mining: Identify the related quantity associated with the sleep parameter of interest.
5. Storing: Store the sleep parameter and associated quantity to a csv file.

Reading: We imported the PSG reports using the NLTK text reading function. The imported text data were stored for the next step.

Tokenizing: We used the NLTK tool to parse the PSG note into sentences and lines, respectively. Such parsing helped to locate the sleep parameters for the extraction and association with the quantity respectively (Figure 1).

Locating: We developed a set of regular expressions to extract the sleep parameters from the corresponding lines received from the NLTK toolkit. These regular expression sets were developed based on the annotated notes by our sleep specialist team. For instance, the TST has been expressed entirely or its abbreviated form, i.e., TST, or a different format completely in some PSG reports (Supplementary Table S2). However, we developed regular expressions to capture common possible formats of the sleep parameters documented in the PSG reports (Supplementary Table S2). The following string patterns were used for each concept: TST: ‘total sleep time’, ‘slept for’, ‘monitored for’, ‘spent for, ‘Total Sleep Time (TST)’; SOL: ‘sleep onset latency’, ‘sleep onset’, ‘latency for’, ‘Sleep Onset Latency (SOL); SE: ‘sleep efficiency’, ‘sleep efficiency for’, ‘Sleep Efficiency (SE)’; WASO: ‘wake after’, ‘wake time after’, ‘total wake’, ‘WASO’, ‘Wake After Sleep Onset (WASO); AHI: ‘apnea hypopnea index’, ‘apnea/hypopnea index’, ‘AHI for’, ‘Apnea Hypopnea Index (AHI)’.

Mining: The nearest neighbor based quantification step extracts the associated quantity for each of the extracted sleep parameters [26]. In some PSG reports, the quantities precede the sleep parameter and vice versa (Supplementary Table S2). The nearest neighbor algorithm selects the nearest quantity as the associated quantity for the sleep parameter of interest, while selecting the second nearest for some cases (Supplementary Table S2). The successive operations of both locating and mining steps associate the sleep parameter of interest with its corresponding quantity [27]. All the information retrieved in this step is forwarded to the next step.

Storing: In this step, the sleep parameter, the associated quantity, patient identifiers, and visit date were stored to a csv file, aka structured data. 

### 2.5. Performance of the NLP Algorithm

The performance of the NLP algorithm has been measured by the accuracy, precision, recall, and F-1 score [28], as this is a standard for performance analysis of the information retrieval algorithm [29]. The accuracy defines the fraction of the relevant information retrieved from all the documents (accuracy = true positive + true negative/total number of notes). The precision defines the fraction of retrieved documents that are in fact relevant (precision = true positive/(true positive + false positive)). The recall defines the fraction of relevant documents that are retrieved by the algorithm (recall = true positive/(true positive + false negative)). The F-1 score measures the combined performance of the recall and precision (F-1 score = 2 × precision × recall/(precision + recall)) [30].

## 3. Results

**Figure 2:**
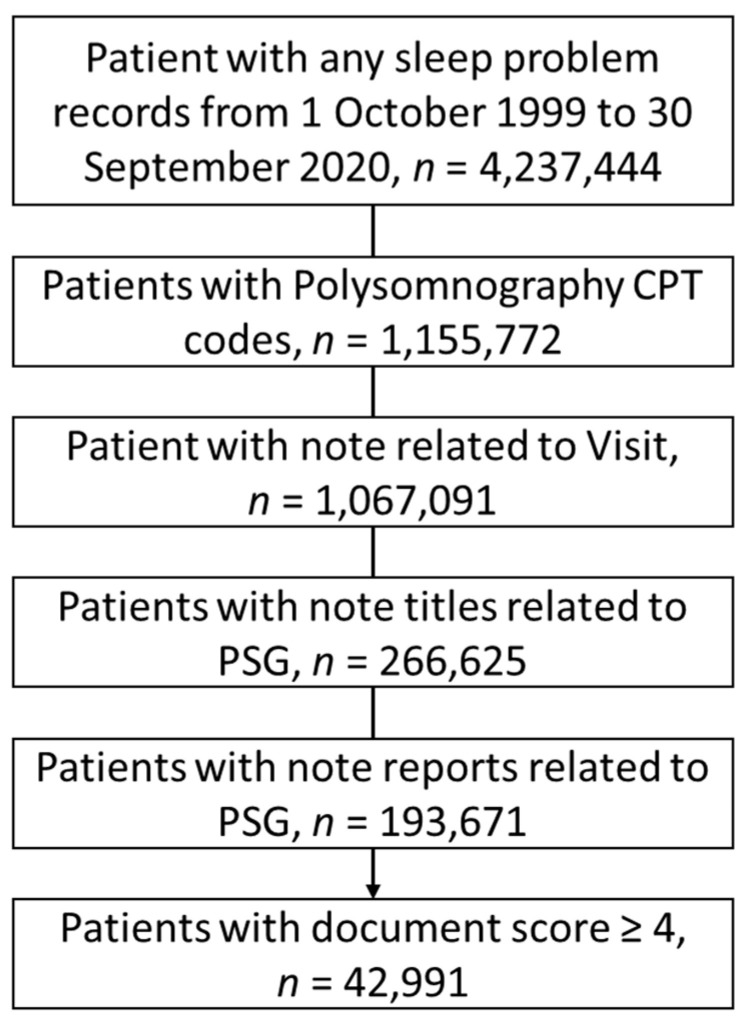
The strobe diagram for collecting patients’ polysomnography reports with CPT codes of ‘95810’, and ‘95811’ who visited veteran health administration (VHA) from 1 October 1999 to 30 September 2020.

In this study, we collected 407,730 PSG notes from 4,237,444 patients who used the VHA for medical sleep advises (88.3% male, 26.3% age ≥ 65 years, 57.7% obese, BMI ≥ 30 Kg/m^2^), Supplementary Table S3 and Supplementary Figure S1. Of 407,730 notes, 46,093 (11.3%) met the DQI criteria ≥ 4, Figure 2. 

### 3.1. Reliability Analysis

The reliability between annotators ranged from 0.51 to 0.89, Table 1. We observed the highest ICC value in SOL (0.89) and the lowest value in WASO (0.51). The ICC values for SE, WASO, and AHI were considered moderate, while the ICC values for TST and SOL were good.

### 3.2. Performance Analysis

We provided the performance analysis for the training and validation datasets in Table 2. In the training phase, the NLP algorithm showed accuracy of 0.91 across all sleep parameters. The highest performance was observed in the SE and WASO, and the lowest performance was observed in the TST (accuracy, 0.91). In the validation phase, SE had the highest performance, i.e.,1.00. The accuracy and recall level of SOL was the lowest (0.90). 

## 4. Discussion

### 4.1. Methods and Results of the NLP Algorithm

In this study, we developed and validated an NLP algorithm to extract sleep parameters from PSG reports stored in the EMR as free-text notes. Additionally, we developed a quality metric to assess whether the PSG report was sufficient quality to be included in data extraction by consulting with a sleep medicine-certified group of physicians (A.S. and R.A.). This is one of the first published studies that used NLP to extract sleep continuity parameters and a respiratory index (AHI) and transform them from unstructured data to structured data. 

In the process of preparing the notes for the NLP algorithm, we excluded notes that reported split nights and performed titration studies. In the split-night PSG, two nights’ recordings are combined into a single overnight observation. The split-night PSG does not provide adequate sleep architecture data for a complete night [31]. We developed an inclusion metric, DQI, that ensured the quality of documentation of the PSG reports. The DQI was developed in-house based on the clinical experiences of our certified sleep medicine physicians (A.S. and R.A.). It provides an additional inclusion layer to ensure quality of the PSG report prior to processing and extracting PSG sleep parameter data. Next, we randomly selected 200 PSG reports to compare accuracy of the NLP algorithm to extract correct sleep parameter data compared to visual inspection by multiple masked raters (JR and MR). We reported moderate-to-good ICC among raters, which originated from variation of expressions in documenting sleep parameter (Supplementary Table S2). Because of variation in documentation, the performance of the NLP also varies. For example, the SE has a regular pattern of number and percentage association that helps to achieve the highest performance (SE sentences in Supplementary Table S2). However, the performance degrades for the anomalies, with either the sleep parameter statement expressed as a different expression or subsequent expression of another sleep parameter (TST, SOL in Table 2). Clinical interpretation and research of PSG reports could benefit by creating a standardized template for sleep reports. Compared to a previous study with average performance of 80% [17], the overall performance of the algorithm to extract sleep parameter is greater than 90%. The previous study developed based on a regular expression approach, while we used a more sophisticated algorithm to extract the sleep parameters [17]. 

### 4.2. Strength and Weakness

There are several limitations of this study. First, we limited the notes to those with proper documentation of PSG reports; and we excluded notes that related to split-night study or home sleep tests. We did this to reduce the variability that may introduce noise when developing and testing the NLP algorithm to extract sleep parameters. However, by doing so we acknowledge we limited the number of notes reviewed. A separate NLP algorithm is warranted to mine the home sleep test notes and extract home sleep test sleep-related respiratory parameters such as AHI and blood oxygen saturation level (SaO2) versus the sleep continuity variables typically found in PSG reports. PSG reports were selected from the VA EMR. Generalizability of the NLP algorithm to extract PSG sleep parameters from other facilities remains to be tested. Finally, future work should develop and test NLP algorithms to extract sleep architecture (Stage N1, REM) and other indices (RDI, Sp02, PLMI).

## 5. Conclusions

We developed and validated an NLP algorithm for systematic extraction of the sleep continuity parameters and a respiratory index (AHI) from the PSG reports. The algorithm has >90% performance in all the performance metrics in both training and validation stages. In recent years there has been a rapid emergence of artificial intelligence (AI) in the field of sleep medicine. AI refers to the capability of computer systems to perform tasks conventionally thought to require human intelligence, such as speech recognition, decision making, and visual recognition of patterns and objects. Additionally, a new advanced machine learning based NLP algorithm such as Bidirectional Encoder Representations from Transformers (BERT) has been introduced developed by Google [32]. Another study is warranted to develop an NLP based on BERT to extract sleep parameters. 

Sleep medicine is well positioned to benefit from advances that use big data to create artificially intelligent computer programs. Leveraging longitudinal data accumulated within EMR, sleep medicine is primed to benefit from AI. As a greater number of sleep studies occur each year within and outside the VHA, utilizing AI and advanced machine learning becomes more appealing to automate data extraction from the EMR [33]. AI techniques will allow clinicians and investigators to examine the wealth of rich, longitudinal real-world data that is housed within the EMR, currently in the form of free-text notes. By using AI techniques, investigators can leverage “big data” to offer new insights into sleep physiology, improve the accuracy of diagnosis of sleep disorders, predict response and adherence to treatment, and use sleep parameters as predictors of future physical and mental health, leading to treatment optimization and personalization.

## Figures and Tables

**Table 1:** The agreement between two raters measured by inter class correlation (95% confidence intervals) on sleep notes.

Sleep Parameter *	N (%)	ICC (95% CI)
TST	195 (97.5)	0.83 (0.78, 0.87)
SE	196 (98.0)	0.59 (0.49, 0.68)
SOL	194 (97.0)	0.89 (0.86, 0.92)
WASO	184 (92.0)	0.51 (0.40, 0.61)
AHI	192 (96.0)	0.62 (0.52, 0.70)

* TST = total sleep time, SE = sleep efficiency, SOL = sleep onset latency, WASO = wake after sleep onset. ICC = inter class correlation.

**Table 2:** Performance analysis of the NLP algorithm.

Sleep Parameter	Accuracy	Precision	Recall	F-1 Score
**Training**				
TST	0.91	0.98	0.93	0.95
SOL	0.91	1.0	0.91	0.96
SE	0.98	1.0	1.0	0.99
WASO	0.98	1.0	0.98	0.99
AHI	0.96	0.99	0.96	0.98
**Validation**				
TST	0.95	1.0	0.95	0.97
SOL	0.90	1.0	0.90	0.95
SE	1.0	1.0	1.0	1.0
WASO	0.95	1.0	0.95	0.97
AHI	0.95	1.0	0.95	0.97

## Data Availability

The dataset is archived at the Corporate Data Warehouse behind the VA firewall and any official request required VA approval.

## Supplementary Materials

The following supporting information can be downloaded at: https://www.mdpi.com/article/10.3390/healthcare10101837/s1, Table S1: list of International Classification of Diseases Clinical Modification (ICD) ninth and tenth edition and Current Procedural Terminology (CPT) code; Table S2: Examples of TST, SE, AHI, WASO, SOL sentences found in the PSG reports; Table S3: Patients' characteristics and demographics; Figure S1: The distribution of sleep report quality aka document quality index (DQI). The DQI is a score ranged from zero to seven. Zero referred to no sleep parameters phrases used in the note and seven referred to full documentation of the sleep parameters. The DQI is calculated by summing seven components and each component related to a sleep parameter as follows: 1) total sleep time, sleep onset latency, sleep efficiency (SE), wake after sleep onset (WASO), Rapid eye movement (REM), sleep stage 1 (N1), and Apnea Hypopnea Index (AHI).
