# Supplementary material for: Validation of a Natural Language Processing Algorithm for the Extraction of the Sleep Parameters from the Polysomnography Reports
Source: Healthcare (Basel). 2022 Sep 22;10(10):1837. doi: 10.3390/healthcare10101837 (PMC9602175; doi:10.3390/healthcare10101837)
Supplement: Supplementary file 1 [file healthcare-10-01837-s001.zip › healthcare-1908855-supplementary.pdf]

Supplement Table S1. list of International Classification of Diseases Clinical Modification (ICD) ninth and tenth edition and Current Procedural Terminology (CPT) code.	
ICD9code	ICD9Description
46.72	FATAL FAMILIAL INSOMNIA
278.03	OBESITY HYPOVENTILATION SYNDROME
283.23	HEMOGLOBINURIA, PAROXYSMAL (COLD) (NOCTURNAL)
291.82	ALCOHOL INDUCED SLEEP DISORDERS
292.85	DRUG INDUCED SLEEP DISORDERS
307.4	NONORGANIC SLEEP DISORDER, UNSPECIFIED
307.41	TRANSIENT DISORDER OF INITIATING OR MAINTAINING SLEEP
307.42	PERSISTENT DISORDER OF INITIATING OR MAINTAINING SLEEP
307.43	TRANSIENT DISORDER OF INITIATING OR MAINTAINING WAKEFULNESS
307.44	PERSISTENT DISORDER OF INITIATING OR MAINTAINING WAKEFULNESS
307.45	CIRCADIAN RHYTHM SLEEP DISORDER
307.45	CIRCADIAN RHYTHM SLEEP DISORDER OF NONORGANIC ORIGIN
307.45	PHASE-SHIFT DISRUPTION OF 24-HOUR SLEEP-WAKE CYCLE
307.46	SLEEP AROUSAL DISORDER
307.46	SOMNAMBULISM OR NIGHT TERRORS
307.47	OTHER DYSFUNCTIONS OF SLEEP STAGES OR AROUSAL FROM SLEEP
307.48	REPETITIVE INTRUSIONS OF SLEEP
307.49	OTHER SPECIFIC DISORDERS OF SLEEP OF NONORGANIC ORIGIN
327	ORGANIC INSOMNIA, UNSPECIFIED
327.01	INSOMNIA DUE TO MEDICAL CONDITION CLASSIFIED ELSEWHERE
327.02	INSOMNIA DUE TO MENTAL DISORDER
327.09	OTHER ORGANIC INSOMNIA
327.1	ORGANIC HYPERSOMNIA, UNSPECIFIED
327.11	IDIOPATHIC HYPERSOMNIA WITH LONG SLEEP TIME
327.12	IDIOPATHIC HYPERSOMNIA WITHOUT LONG SLEEP TIME
327.13	RECURRENT HYPERSOMNIA
327.14	HYPERSOMNIA DUE TO MEDICAL CONDITION CLASSIFIED ELSEWHERE
327.15	HYPERSOMNIA DUE TO MENTAL DISORDER
327.19	OTHER ORGANIC HYPERSOMNIA
327.2	ORGANIC SLEEP APNEA, UNSPECIFIED
327.21	PRIMARY CENTRAL SLEEP APNEA
327.22	HIGH ALTITUDE PERIODIC BREATHING
327.23	OBSTRUCTIVE SLEEP APNEA (ADULT) (PEDIATRIC)
327.24	IDIOPATHIC SLEEP RELATED NON-OBSTRUCTIVE ALVEOLAR HYPOVENTILATION
327.25	CONGENITAL CENTRAL ALVEOLAR HYPOVENTILATION SYNDROME
327.26	SLEEP RELATED HYPOVENTILATION/HYPOXEMIA IN CONDITIONS CLASSIFIABLE ELSEWHERE
327.27	CENTRAL SLEEP APNEA IN CONDITIONS CLASSIFIED ELSEWHERE

327.29	OTHER ORGANIC SLEEP APNEA
327.3	CIRCADIAN RHYTHM SLEEP DISORDER, UNSPECIFIED
327.31	CIRCADIAN RHYTHM SLEEP DISORDER, DELAYED SLEEP PHASE TYPE
327.32	CIRCADIAN RHYTHM SLEEP DISORDER, ADVANCED SLEEP PHASE TYPE
327.33	CIRCADIAN RHYTHM SLEEP DISORDER, IRREGULAR SLEEP-WAKE TYPE
327.34	CIRCADIAN RHYTHM SLEEP DISORDER, FREE-RUNNING TYPE
327.35	CIRCADIAN RHYTHM SLEEP DISORDER, JET LAG TYPE
327.36	CIRCADIAN RHYTHM SLEEP DISORDER, SHIFT WORK TYPE
327.37	CIRCADIAN RHYTHM SLEEP DISORDER IN CONDITIONS CLASSIFIED ELSEWHERE
327.39	OTHER CIRCADIAN RHYTHM SLEEP DISORDER
327.4	ORGANIC PARASOMNIA, UNSPECIFIED
327.41	CONFUSIONAL AROUSALS
327.42	REM SLEEP BEHAVIOR DISORDER
327.43	RECURRENT ISOLATED SLEEP PARALYSIS
327.44	PARASOMNIA IN CONDITIONS CLASSIFIED ELSEWHERE
327.49	OTHER ORGANIC PARASOMNIA
327.51	PERIODIC LIMB MOVEMENT DISORDER
327.52	SLEEP RELATED LEG CRAMPS
327.53	SLEEP RELATED BRUXISM
327.59	OTHER ORGANIC SLEEP RELATED MOVEMENT DISORDERS
327.8	OTHER ORGANIC SLEEP DISORDERS
333.94	RESTLESS LEGS SYNDROME
347	CATAPLEXY AND NARCOLEPSY
347	NARCOLEPSY, WITHOUT CATAPLEXY
347.01	NARCOLEPSY, WITH CATAPLEXY
347.1	NARCOLEPSY IN CONDITIONS CLASSIFIED ELSEWHERE, WITHOUT CATAPLEXY
347.11	NARCOLEPSY IN CONDITIONS CLASSIFIED ELSEWHERE, WITH CATAPLEXY
368.16	PSYCHOPHYSICAL VISUAL DISTURBANCES
495.7	VENTILATION PNEUMONITIS
770.81	PRIMARY APNEA OF NEWBORN
770.82	OTHER APNEA OF NEWBORN
780.5	UNSPECIFIED SLEEP DISTURBANCE
780.51	INSOMNIA WITH SLEEP APNEA,UNSPECIFIED
780.51	INSOMNIA WITH SLEEP APNEA
780.52	OTHER INSOMNIA
780.52	INSOMNIA, UNSPECIFIED
780.53	HYPERSOMNIA WITH SLEEP APNEA
780.53	HYPERSOMNIA WITH SLEEP APNEA,UNSPECIFIED
780.54	HYPERSOMNIA, UNSPECIFIED

780.54	OTHER HYPERSOMNIA
780.55	DISRUPTION OF 24 HOUR SLEEP WAKE CYCLE, UNSPECIFIED
780.55	DISRUPTIONS OF 24-HOUR SLEEP-WAKE CYCLE
780.56	DYSFUNCTIONS ASSOCIATED WITH SLEEP STAGES OR AROUSAL FROM SLEEP
780.57	UNSPECIFIED SLEEP APNEA
780.57	OTHER AND UNSPECIFIED SLEEP APNEA
780.58	SLEEP RELATED MOVEMENT DISORDER, UNSPECIFIED
780.58	SLEEP RELATED MOVEMENT DISORDER
780.59	OTHER SLEEP DISTURBANCES
786.01	HYPERVENTILATION
786.03	APNEA
788.36	NOCTURNAL ENURESIS
E002.6	ACTIVITIES INVOLVING WATER SKIING AND WAKE BOARDING
V69.4	LACK OF ADEQUATE SLEEP
V69.5	BEHAVIORAL INSOMNIA OF CHILDHOOD
ICD10code	ICD10Description
A81.83	Fatal familial insomnia
D59.5	Paroxysmal nocturnal hemoglobinuria [Marchiafava-Micheli]
E66.2	Morbid (severe) obesity with alveolar hypoventilation
F10.182	Alcohol abuse with alcohol-induced sleep disorder
F10.282	Alcohol dependence with alcohol-induced sleep disorder
F10.982	Alcohol use, unspecified with alcohol-induced sleep disorder
F11.182	Opioid abuse with opioid-induced sleep disorder
F11.282	Opioid dependence with opioid-induced sleep disorder
F11.982	Opioid use, unspecified with opioid-induced sleep disorder
F13.182	Sedative, hypnotic or anxiolytic abuse with sedative, hypnotic or anxiolytic-induced sleep disorder
F13.282	Sedative, hypnotic or anxiolytic dependence with sedative, hypnotic or anxiolytic-induced sleep disorder
F13.982	Sedative, hypnotic or anxiolytic use, unspecified with sedative, hypnotic or anxiolytic-induced sleep disorder
F14.182	Cocaine abuse with cocaine-induced sleep disorder
F14.282	Cocaine dependence with cocaine-induced sleep disorder
F14.982	Cocaine use, unspecified with cocaine-induced sleep disorder
F15.182	Other stimulant abuse with stimulant-induced sleep disorder
F15.282	Other stimulant dependence with stimulant-induced sleep disorder
F15.982	Other stimulant use, unspecified with stimulant-induced sleep disorder
F19.182	Other psychoactive substance abuse with psychoactive substance-induced sleep disorder
F19.282	Other psychoactive substance dependence with psychoactive substance-induced sleep disorder
F19.982	Other psychoactive substance use, unspecified with psychoactive substance-induced sleep disorder

F51.01	Primary insomnia
F51.02	Adjustment insomnia
F51.03	Paradoxical insomnia
F51.04	Psychophysiologic insomnia
F51.05	Insomnia due to other mental disorder
F51.09	Other insomnia not due to a substance or known physiological condition
F51.11	Primary hypersomnia
F51.12	Insufficient sleep syndrome
F51.13	Hypersomnia due to other mental disorder
F51.19	Other hypersomnia not due to a substance or known physiological condition
F51.3	Sleepwalking [somnambulism]
F51.4	Sleep terrors [night terrors]
F51.5	Nightmare disorder
F51.8	Other sleep disorders not due to a substance or known physiological condition
F51.9	Sleep disorder not due to a substance or known physiological condition, unspecified
F52.22	Female sexual arousal disorder
G25.81	Restless legs syndrome
G47.00	Insomnia, unspecified
G47.01	Insomnia due to medical condition
G47.09	Other insomnia
G47.10	Hypersomnia, unspecified
G47.11	Idiopathic hypersomnia with long sleep time
G47.12	Idiopathic hypersomnia without long sleep time
G47.13	Recurrent hypersomnia
G47.14	Hypersomnia due to medical condition
G47.19	Other hypersomnia
G47.20	Circadian rhythm sleep disorder, unspecified type
G47.21	Circadian rhythm sleep disorder, delayed sleep phase type
G47.22	Circadian rhythm sleep disorder, advanced sleep phase type
G47.23	Circadian rhythm sleep disorder, irregular sleep wake type
G47.24	Circadian rhythm sleep disorder, free running type
G47.25	Circadian rhythm sleep disorder, jet lag type
G47.26	Circadian rhythm sleep disorder, shift work type
G47.27	Circadian rhythm sleep disorder in conditions classified elsewhere
G47.29	Other circadian rhythm sleep disorder
G47.30	Sleep apnea, unspecified
G47.31	Primary central sleep apnea
G47.32	High altitude periodic breathing
G47.33	Obstructive sleep apnea (adult) (pediatric)
G47.34	Idiopathic sleep related nonobstructive alveolar hypoventilation
G47.35	Congenital central alveolar hypoventilation syndrome

G47.36	Sleep related hypoventilation in conditions classified elsewhere
G47.37	Central sleep apnea in conditions classified elsewhere
G47.39	Other sleep apnea
G47.411	Narcolepsy with cataplexy
G47.419	Narcolepsy without cataplexy
G47.421	Narcolepsy in conditions classified elsewhere with cataplexy
G47.429	Narcolepsy in conditions classified elsewhere without cataplexy
G47.50	Parasomnia, unspecified
G47.51	Confusional arousals
G47.52	REM sleep behavior disorder
G47.53	Recurrent isolated sleep paralysis
G47.54	Parasomnia in conditions classified elsewhere
G47.59	Other parasomnia
G47.61	Periodic limb movement disorder
G47.62	Sleep related leg cramps
G47.63	Sleep related bruxism
G47.69	Other sleep related movement disorders
G47.8	Other sleep disorders
G47.9	Sleep disorder, unspecified
H53.16	Psychophysical visual disturbances
H93.241	Temporary auditory threshold shift, right ear
H93.242	Temporary auditory threshold shift, left ear
H93.243	Temporary auditory threshold shift, bilateral
H93.249	Temporary auditory threshold shift, unspecified ear
N39.44	Nocturnal enuresis
P28.3	Primary sleep apnea of newborn
P28.4	Other apnea of newborn
R06.3	Periodic breathing
R06.4	Hyperventilation
R06.81	Apnea, not elsewhere classified
R45.1	Restlessness and agitation
Y93.17	Activity, water skiing and wake boarding
Y93.84	Activity, sleeping
Z72.820	Sleep deprivation
Z72.821	Inadequate sleep hygiene
Z73.810	Behavioral insomnia of childhood, sleep-onset association type
Z73.811	Behavioral insomnia of childhood, limit setting type
Z73.812	Behavioral insomnia of childhood, combined type
Z73.819	Behavioral insomnia of childhood, unspecified type
CPTCode	CPTDescription

95810	POLYSOMNOGRAPHY; AGE 6 YEARS OR OLDER, SLEEP STAGING WITH 4 OR MORE ADDITIONAL PARAMETERS OF SLEEP, ATTENDED BY A TECHNOLOGIST
95811	POLYSOMNOGRAPHY; AGE 6 YEARS OR OLDER, SLEEP STAGING WITH 4 OR MORE ADDITIONAL PARAMETERS OF SLEEP, ATTENDED BY A TECHNOLOGIST AIRWAY PRESSURE THERAPY OR BILEVEL VENTILATION, ATTENDED BY A TECHNOLOGIST

Table S2. Examples of TST, SE, AHI, WASO, SOL sentences found in the PSG reports.

TST Sentence	SE Sentence	AHI Sentence	WASO Sentence	SOL Sentence

**The total sleep time is 350 min.**	Total sleep time was 276.5 minutes resulting in a **sleep efficiency** of **63.0**%.	The **Apnea Hypopnea Index** through the night was **14.5** hr.	**Wakefulness** after sleep onset **WASO**: **19.5** min.	The **sleep latency** and stage N2 were **11.0** and 6.0 min.
**260 min is TST.**	305 minutes TST **85.2**% **SE.**	Apnea Hypopneas: 176 **Apnea Hypopnea Index AHI** events hr sleep: **27.7.**	68 min **Wake** after S O **WASO**.	**Latency to stage** N1 sleep was **16.0** min and latency to REM from **sleep onset** was 71.0 min.
**The patient spent 400 min in sleep.**	Sleep onset latency was very delayed at 312 minutes and **sleep efficiency** was very poor at **18**%.	No obstructive hypopneas recorded during this study for an **apnea hypopnea index AHI 0.2** events per hour.	Total Apneas: 15 **Total Wake Time** min.	**Sleep onset** occurred at 10:17:01 PM for a **sleep latency** of **6.7** min.
**The patient was monitored for 357 minutes with 297 minutes of sleep.**	The Time in Bed Sleep Period Time Total Sleep Time and **Sleep Efficiency** were 414.5 min 369.5 min 305.5 min and **77.6**% respectively.	There were 0 apneas and 11 hypopneas resulting in an apnea hypopnea index mean number of **apneas and hypopneas per hour** of sleep of **2.3** normal 5.	**Total Wake time** min: 133.5.	**Sleep latency** 10.0 min.

Table S3. Patients' characteristics and demographics.

Sex, Male, N(%)	37284(88.3)
Death, N(%)	8018(19.0)

Race-White, N(%)	30982(73.4)
Race-Black, N(%)	8940(21.2)
Race-Others, N(%)	2291(5.4)
Age, M(SD)	53.9(14.7)
Age, <30, N(%)	2508(5.9)
Age, 30-40, N(%)	6008(14.2)
Age, 40-50, N(%)	7108(16.8)
Age, 50-65, N(%)	15517(36.8)
Age, 65-75, N(%)	8346(19.8)
Age, ≥75, N(%)	2726(6.5)
BMI, M(SD)	31.8(6.1)
BMI ≥ 30, N(%)	24374(57.7)

M(SD) = mean and standard deviation, BMI = Body Mass Index

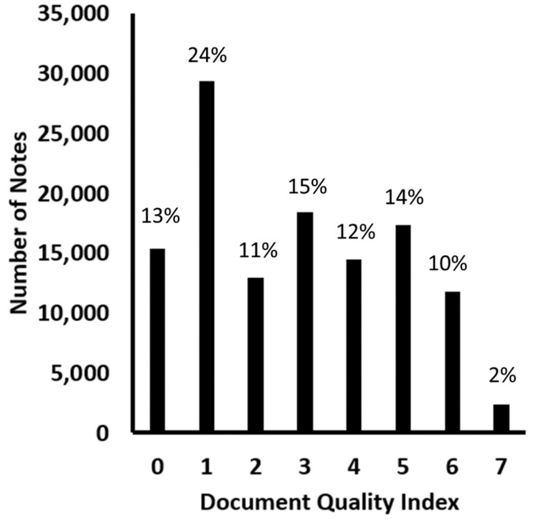

Figure S1: The distribution of sleep report quality aka document quality index (DQI). The DQI is a score ranged from zero to seven. Zero referred to no sleep parameters phrases used in the note and seven referred to full documentation of the sleep parameters. The DQI is calculated by summing seven components and each component related to a sleep parameter as follows: 1) total sleep time, sleep onset latency, sleep efficiency (SE), wake after sleep onset (WASO), Rapid eye movement (REM), sleep stage 1 (N1), and Apnea Hypopnea Index (AHI).
